# Sulfur and Its Derivatives in Dermatology: Insights Into Therapeutic Applications—A Narrative Review

**DOI:** 10.1111/jocd.70402

**Published:** 2025-08-15

**Authors:** Yan Jing Chen, Jie Tang, Lin Wang, Wei Hua

**Affiliations:** ^1^ Department of Dermatology and Venereology West China Hospital, Sichuan University Chengdu China; ^2^ Cosmetics Safety and Efficacy Evaluation Center, West China Hospital, Sichuan University Chengdu Sichuan China; ^3^ Sichuan Engineering Technology Research Center of Cosmetic Chengdu Sichuan China

**Keywords:** hydrogen sulfide, selenium disulfide, sulfur

## Abstract

**Background:**

Sulfur has been historically used in dermatological therapy due to its broad‐spectrum antimicrobial and immunomodulatory activities and demonstrates therapeutic efficacy in conditions such as scabies, tinea versicolor, psoriasis, and atopic dermatitis. However, systematic analyses of the therapeutic potential and mechanisms of different forms of sulfur (e.g., sublimed and precipitated sulfur) and bioactive derivatives (e.g., hydrogen sulfide), particularly their effects on cutaneous diseases, remain underrepresented in contemporary literature.

**Aim:**

Our study aimed to provide a comprehensive evaluation of pharmacological evidence and regulatory frameworks governing sulfur and its derivatives in dermatology, elucidating their mechanisms and therapeutic potential in various skin disorders.

**Methods:**

A comprehensive PubMed search was conducted using medical terms including sulfur, sulfide, hydrogen sulfide, and dermatology. Relevant literature published between 1947 and 2025 was reviewed.

**Results:**

This article not only summarizes the indications, potential therapeutic value, and mechanisms of sulfur and its derivatives in skin disorders, but also provides, for the first time, an overview of the usage restrictions and regulations established by food and drug administrations in most regions regarding the application of sulfur and its derivatives in dermatology.

**Conclusions:**

This review highlights critical gaps in understanding the therapeutic bioactivity of sulfur and its derivatives, underscoring the need for preclinical studies to explore their translational potential as adjuvant therapies in dermatology.

## 1 Introduction

**FIGURE 1:**
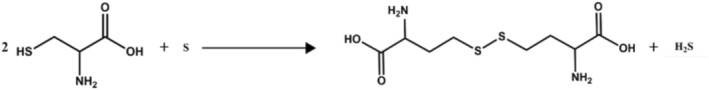
Sulfur and its possible interaction with the cysteine content of keratinocytes.

Sulfur, a nonmetal element, has a historical medicinal use dating back to Hippocrates for treating plague [1, 2]. In dermatology, it has been widely used to treat dermatoses such as acne, rosacea, seborrheic dermatitis, scabies, and tinea versicolor [2]. Sulfur was reported to have keratoplastic (low concentration), keratolytic (higher concentration), and antifungal effects when applied topically to the skin [2]. When sulfur interacts with the skin, a chemical reaction occurs in keratinocytes between cysteine and sulfur, producing cystine and hydrogen sulfide (H_2_S) (Figure 1) [2]. Previous studies indicate that H_2_S has a crucial function in vasodilation, wound healing, inflammation, antioxidation, and neoplastic cell regulation [3]. However, various factors influence the function of H_2_S, including its concentration, reaction time, and the types of cells or diseases involved [4].

Sulfur exhibits valence states ranging from +6 to −2 and stable oxidation states, forming diverse inorganic compounds—including elemental sulfur, sulfate (SO_4_^2−^), sulfide (S^2−^), sulfite (SO_3_^2−^), thiosulfate (S_2_O_3_^2−^), and polythionates (S_3_O_6_^2−^, S_4_O_6_^2−^)^5^—as well as organosulfur derivatives such as thiosulfonate [1, 5]. In dermatological formulations, precipitated sulfur, with its smaller particle size, is therapeutically superior than sublimed sulfur, which exists in bar soaps, shampoos, gels, lotions, and creams [2, 6].

**FIGURE 2:**
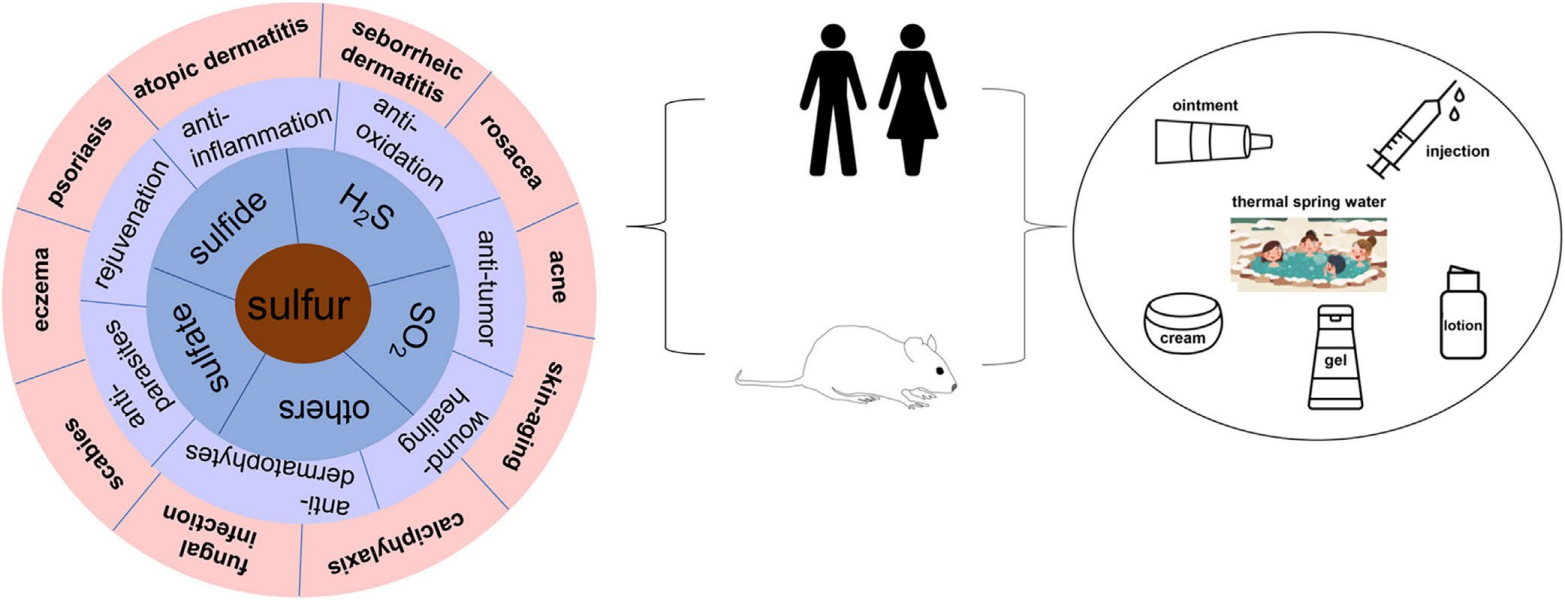
Sulfur and its derivatives in dermatology.

This overview presents the underlying mechanisms and applications of sulfur and its derivatives in the treatment of different dermatological diseases (Figure 2).

Possible mechanism of sulfur and its derivatives in improving skin disorders (Table 1).

1. The formation of cystine might promote normal keratinization, which results from the chemical reaction between sulfur and the skin [2].
2. Dissolution of the stratum corneum might happen when a high concentration of sulfur is applied to the skin, caused by sufficient accumulation of H_2_S that breaks down the bonds between keratins [2].
3. Toxicity to fungi might be achieved through the conversion to pentathionic acid when sulfur was applied to the skin [2]. On the other hand, protein precipitation and denaturation occur when sulfide salts react with dermatophytic enzymes [14].
4. H_2_S exerts anti‐inflammatory activity by interacting with important inflammatory signaling pathways such as NF‐κB and nuclear factor‐erythroid 2‐related factor 2 (Nrf2) [7].
5. Inhibition of T‐cell proliferation and inflammatory cytokine production (e.g., IL‐2, TNF‐α, IL‐6, IL‐8, nitric oxide (NO), IFN‐γ, and CXCL‐2) by sulfur and H_2_S might contribute to the anti‐inflammation process [8, 9, 10].
6. H_2_S has antioxidant effects by increasing reduced glutathione and directly scavenging superoxide anions, hydrogen peroxide, and peroxynitrite. It protects neurons and inhibits the proliferation and activity of human lymphocytes [11].
7. Apoptosis can be induced by cytotoxic sulfur dioxide (SO_2_), accompanied by an increase in intracellular reactive oxygen species (ROS) levels and DNA damage in tumor cells [15]. H_2_S exerts anticancer effects that might be related to the inhibition of NF‐κB activation, a decrease in AKT and ERK phosphorylation, and the suppression of the PI3K/AKT/mTOR and integrin/FAK pathways [3].
8. Targeting H_2_S delivery to mitochondria may have antisenescence effects, it suppresses cytotoxicity induced by UVA, decreases matrix metalloproteases (MMP)‐1 activity, and increases the expression of collagen and Nrf2 levels [12].
9. By increasing the production of vascular endothelial growth factor (VEGF), epidermal growth factor (EGF), platelet‐derived growth factor (PDGF), hypoxia‐inducible factor‐1α (HIF‐1α), and endothelial nitric oxide synthase (eNOS), H_2_S may promote diabetic wound healing [13].

**TABLE 1:** The role of sulfur and its derivatives in the treatment of skin disorders.

Functional component	Function
Sulfur	Promote normal keratinization [2]
H_2_S	Exfoliate the stratum corneum [2, 7]
	Reduce skin inflammation [3, 7, 8, 9, 10]
	Antioxidation [7, 11]
	Protect neurons [11]
	Exert anticancer effects [3]
	Antiaging effects [12]
	Promote wound healing [13]
Pentathionic acid Sulfide salt	Damage dermatophyte [7, 14]
SO_2_	Induce apoptosis of tumor cells [15]

Abbreviations: H_2_S, hydrogen sulfide; SO_2_, sulfur dioxide.

Applications of sulfur and its derivatives in different dermatological diseases (Table 2).

**TABLE 2:** Applications of sulfur and derivatives in dermatology.

Function	Skin disorders	Strength
Antiscabetic	Scabies	2%–12.5%
Antifungal	Tinea versicolor, tinea capitis	2.5%
	Seborrheic dermatitis	1%–2%
Anti‐inflammatory	Atopic dermatitis, psoriasis	—
	Psoriasis	30 mg/mL
	Psoriasis, eczema, seborrheic dermatitis	4%
	Eczema, acne, seborrheic dermatitis	200 mg/g
	Acne, rosacea	5%
	Hand eczema	2%
Vasodilation	Calciphylaxis	125 mg/mL
		250 mg/mL
Rejuvenating	Skin‐aging	—
Wound healing	Chronic wounds	2%
		6%, 12%

Formulation
Sulfur cream/ointment (sublimed/precipitated sulfur)
Selenium sulfide lotion (sulfide salt)
Zinc pyrithione (thiol)
Sulfur thermal spring water (sulfur, H_2_S, and sulfates)
Psor‐assist cream (precipitated sulfur)
Coco‐scalp ointment (coal tar 12%, salicylic acid 2%, precipitated sulfur 4%)
Zinc oxide and sulfur ointment (sublimed sulfur)
Sodium sulfacetamide/sulfur cream/cleanser
Sulfur cream
Sodium thiosulfate injection (inorganic sulfur)
AP39, AP123 (H_2_S donors)
NaHS‐containing ointment (H_2_S)
Sodium thiosulfate gels

Abbreviations: H_2_S, hydrogen sulfide; NaHS, sodium hydrosulfide.

## 2 In Infectious Skin Diseases

Sarcoptes scabiei var. hominis causes scabies, a parasitic skin disease. It usually manifests intense pruritus and erythematous papules, predominantly in interdigital spaces of the fingers, wrists, and genital regions [16]. Sulfur is the oldest antiparasitic agent in use. Topical cream or ointment (2%–12.5%) was used in treating scabies [17]. Sulfur may dislodge scabies mites from the stratum corneum due to its keratolytic effects [2]. However, the precise mechanism underlying sulfur's antiparasitic activity has not been clarified. Furthermore, there is a lack of convincing clinical evidence supporting sulfur's superior efficacy over permethrin and ivermectin [18].

Tinea versicolor usually presents clinically with hypopigmented or hyperpigmented macules on the face, arms, and trunk, which is caused by Malassezia fungal infection. In vitro, selenium sulfide forms complexes with dermatophytic enzymes that precipitate and denature proteins [14]. Topical selenium sulfide (2.5%) and zinc pyrithione are effective in managing Tinea versicolor [19, 20].

## 3 In Inflammatory Diseases

Perioral dermatitis, which commonly occurs in children and females, is characterized by papulopustules and vesicles, accompanied by pruritus or pain. Topical sodium sulfacetamide lotion has been reported as part of a combination treatment for perioral dermatitis, but its effects remain controversial [21].

Hand eczema affects a large population and causes severe psychosocial burden. Patients with hand eczema may present with polymorphous lesions including infiltrated papules, plaques, vesicles, fissures, edema, or scaling. A randomized, triple‐blind clinical trial that recruited 70 patients with chronic hand eczema showed that 2% sulfur cream was as effective as 0.1% triamcinolone after 4 weeks of treatment, and no adverse effects were reported [22].

Atopic dermatitis (AD) is a recurrent, chronic, noninfectious inflammatory dermatosis characterized by persistent itching of the skin. It manifests as erythema, papules, and exudative lesions in specific locations [23]. Currently, balneotherapy is being practiced in many countries. Spring water is generally rich in sulfur, H_2_S, and sulfates, and thermal sulfur water exerts beneficial anti‐inflammatory and antipruritic effects [24]. It is widely used to treat psoriasis and AD and has been reported to achieve a high rate of improvement [24, 25]. However, the chemical composition of spring water varies [24]. Sulfur‐containing spring water has been reported to inhibit T‐cell proliferation in a dose‐dependent manner and reduce inflammatory cytokine production. Additionally, H_2_S plays a critical role in the anti‐inflammatory action by interacting with major inflammatory signaling pathways—NF‐κB and Nrf2 [7, 8]. Sodium hydrosulfide (NaHS), a donor of H_2_S, significantly attenuated inflammatory responses in the lesional skin of wild‐type mice [9]. However, how sulfur exerts its effects on various inflammatory skin disorders remains to be explored.

Regarding the antipruritic effects of H_2_S and sulfur, its impacts were controversial in AD patients. Type 2 protease‐activated receptors (PAR‐2) largely mediate the pruritus in AD [26]. NaHS significantly reduces pruritus induced by PAR‐2 activation in mice [27]. However, *Moniaga's* study found that H_2_S‐producing enzymes were increased in human AD skin lesions, and H_2_S donor stimulation upregulated nerve elongation factors (including nerve growth factor and artemin) while downregulating nerve repulsion factor, such as semaphoring 3A levels, in cultured normal human epidermal keratinocytes. This is considered a cause of peripheral itch sensitization in AD skin lesions and suggests that H_2_S partially modulates epidermal hyperinnervation in human skin [4]. However, more research into this phenomenon is needed. Clinically, some trials have demonstrated the positive effects of spring water on AD. A large cohort study recruiting 867 children proved that thermal spring water balneotherapy has safe and beneficial characteristics as a complementary therapy for pediatric AD patients [28].

Psoriasis, a T‐lymphocyte‐driven chronic inflammatory disorder, manifests clinically as well‐demarcated erythematous plaques with adherent silver‐white scales, predominantly localized to extensor surfaces and scalp. In an in vitro study revealed H_2_S exerts dose‐dependent inhibition on TNF‐α‐induced upregulation of NO, IL‐6, and IL‐8 in HaCaT human keratinocytes [10]. Sulfide water was inferred to be helpful in psoriasis due to its enhanced penetration of minerals and positive effects on the skin including keratolytic, antimicrobial, and immunomodulatory properties [29, 30]. A study involving 12 Caucasian patients with mild‐to‐moderate plaque psoriasis demonstrated that sulfur‐rich mineral water (pH 6.85) improved disease severity in psoriasis patients [30]. However, not all spring water is beneficial. A sulfur spring in northern Taiwan with strong acidity was reported to cause acute irritative contact dermatitis, which later developed into scars in a healthy man [31]. The pH of sulfurous mineral water is essential for its therapeutic effects. At skin pH (4.5–6.5), virtually all sulfur is present as H_2_S [32]. It is noteworthy that patients should be aware of the chemical composition, pH, and temperature of spring water to avoid skin irritation and itching when considering this treatment regimen.

Acne is an inflammatory disease with worldwide prevalence, primarily affecting adolescents [33]. Sulfur still plays a vital role in the management of pustular acne, despite the availability of more advanced alternatives for treating acne lesions [2]. A study by Draelos et al. was conducted to evaluate the safety and efficacy of an emollient foam formulation containing 10% sodium sulfacetamide and 5% sulfur in treating rosacea, seborrheic dermatitis, and acne vulgaris [34]. Sulfur is effective in these conditions due to its keratolytic effect, caused by breaking disulfide bonds between corneocytes, and its antibacterial activity [32]. In a retrospective study, 300 patients with seborrheic dermatitis, acne, and rosacea used a compounded topical agent (hydrocortisone 0.75% and precipitated sulfur 0.5%) [35]. Moreover, nano‐sized sulfur has presented new possibilities for acne vulgaris resistant to conventional antibiotics [36]. Currently, topical sulfur‐containing agents (e.g., 20% sublimed sulfur) remain available for off‐label use in treating acne vulgaris, though evidence supporting their efficacy is limited. Common cooccurrence dermatoses, such as seborrheic dermatitis, may improve with sulfide‐containing agents.

Rosacea is a chronic, relapsing, inflammatory dermatosis that affects up to 10% of the world's population. It has different phenotypes like persistent erythema, facial papules, and pustules. Sodium sulfacetamide/sulfur (10% and 5%) has been proven to be effective in relieving skin erythema and inflammatory lesions in rosacea patients and was superior to metronidazole (0.75%) [37, 38]. Similarly, supportive evidence for topical sulfur's efficacy is scarce in this application.

## 4 In Skin Photoaging

Skin aging manifests as wrinkling, loss of elasticity, laxity, and a rough‐textured appearance [39]. The activation of MMPs and the subsequent degradation of collagen result in skin photoaging, which significantly correlates with oxidative stress and mitochondrial dysfunction. AP39 and AP123, novel mitochondria‐targeted H_2_S delivery molecules, could suppress UVA‐induced cytotoxicity, decrease MMP‐1 activity, and increase collagen expression and Nrf2 levels in vitro. Notably, targeting H_2_S delivery to mitochondria also demonstrated a preventive effect on mouse skin [12]. Hence, investigations into targeting H_2_S delivery for the prevention and treatment of skin photoaging might be valuable.

## 5 In Chronic Wounds

According to previous studies, H_2_S could increase the production of VEGF, EGF, PDGF, HIF‐1α, and eNOS, leading to the healing of diabetic wounds [13]. Topical treatment with a 2% NaHS‐containing ointment and sodium thiosulfate gels (6% and 12%) has been proven to be effective in wound healing in rats [40, 41]. However, explorations of such topical agents applied to the human body are needed. Skin disorders such as pressure ulcers and epidermal cysts following incision and drainage warrant further investigation. Calciphylaxis, commonly seen in patients with end‐stage renal disease, manifests as livedo reticularis, plaques, nodules, and ulcers, which are caused by the occlusion of cutaneous blood vessels [42]. Sodium thiosulfate (STS) may serve as an antioxidant and vasodilator that prevents calcification of blood vessels. Intravenous STS and intralesional STS are optional treatments for calciphylaxis‐associated ulcers, though their use remains off‐label [43]. The main adverse effects include nausea and vomiting [42].

## 6 Complications

Bad odor and xerotic eczema are the most common side effects [17]. Other adverse effects include local irritation, contact allergy, or sulfur spring dermatitis [20, 31]. Topical products with sulfur concentrations less than 6% are potentially safe for use in children (> 2 months), pregnant women, and lactating individuals [17]. The application of sulfur‐containing products to body surface areas has not been demonstrated. Approval and regulations regarding the dermatological use of sulfur and its derivatives can be found in Tables S1 and S2.

## 7 Conclusion

Sulfur and its derivatives exhibit diverse therapeutic effects across various skin disorders. Despite their historical use and proven benefits, challenges remain in understanding their mechanisms across various skin disorders and developing effective formulations. Further research and clinical trials are necessary to optimize sulfur‐based therapies in dermatology.

## Author Contributions

Y.J.C., J.T: conceptualization and literature research and writing – original draft. L.W., W.H.: writing – review and editing. Y.J.C., W.H.: figure generation/visualization. All authors have read and agreed to the version of the manuscript.

## Ethics Statement

The authors have nothing to report.

## Conflicts of Interest

The authors declare no conflicts of interest.

## Supporting information


**Tables S1–S2:** jocd70402‐sup‐0001‐TablesS1‐S2.docx.

## Data Availability

The data that support the findings of this study are available from the corresponding author upon reasonable request.
