# Supplementary material for: Sulfur and Its Derivatives in Dermatology: Insights Into Therapeutic Applications—A Narrative Review
Source: J Cosmet Dermatol. 2025 Aug 15;24(8):e70402. doi: 10.1111/jocd.70402 (PMC12355337; doi:10.1111/jocd.70402)
Supplement: Supplementary file 1 — Tables S1–S2: jocd70402‐sup‐0001‐TablesS1‐S2.docx. [file JOCD-24-e70402-s001.docx]

**Supplementary Table 1 Approval of sulfur-based drugs & OTC products (FDA, NMPA, PMDA, TGA)**

| **Drug name** | **Strength** | **Dosage form** |
| --- | --- | --- |
| Selenium disulfide | 2.5% | lotion |
| Sodium thiosulfate injection | 125 mg/ml | injection |
| Sodium thiosulfate injection | 250 mg/ml | injection |
| Sodium thiosulfate injection | 0.5g/10ml 1g/20ml 1g/4ml 10g/20ml | injection |
| Sodium thiosulfate hydrate | 2g/20ml | injection |
| Zinc oxide and sulfur ointment | 20% | ointment |
| Zinc oxide and sulfur sublimate ointment | 7% | ointment |
| Dyclonine hydrochloride, chlorhexidine acetate and sublimed sulfur ointment | 10% | ointment |
| Compound sulfur cream | 3% | cream |
| Sulfur ointment | 10% | ointment |
| Sulfur cream/suspension/emulsion | 3-10% | cream/suspension/emulsion |
| Sulfur and camphor lotion | 6% | lotion |
| Psor-asist cream jar | 30 mg/ml | cream |
| Coco-scalp ointment | 4% | ointment |

FDA= U.S. Food and Drug Administration; NMPA= National Medical Products Administration (China); PMDA= Pharmaceuticals and Medical Devices Agency (Japan); TGA= Therapeutic Goods Administration (Australia); OTC= Over-the-Counter.

**Supplementary Table 1 Approval of sulfur-based drugs & OTC products (FDA, NMPA, PMDA, TGA)**

| **Route** | **Indication** |
| --- | --- |
| topical | seborrheic dermatitis,  tinea versicolor,  tinea capitis |
| intravenous | reduce  cisplatin-induced ototoxicity in pediatric solid tumors |
| intravenous | acute cyanide poisoning |
| intravenous | cyanide poisoning arsenic, mercury, lead, bismuth, iodine and other poisonings |
| intravenous | cyanide and cyanide compound poisoning,  arsenic compound poisoning |
| topical | scabies, eczema |
| topical | acne,  rosacea,  sebborheic dermatitis |
| topical | scabies |
| topical | sebborheic dermatitis,  scabies,  acne,  eczema |
| topical | scabies,  tinea capitis,  acne,  sebborheic dermatitis,  rosacea,  eczema |
| topical | scabies,  tinea,  psoriasis,  acne,  seborrhea,  chronic eczema |
| topical | acne,  rosacea |
| topical | psoriasis |
| topical | psoriasis,  eczema,  sebborheic dermatitis |

FDA= U.S. Food and Drug Administration; NMPA= National Medical Products Administration (China); PMDA= Pharmaceuticals and Medical Devices Agency (Japan); TGA= Therapeutic Goods Administration (Australia); OTC= Over-the-Counter.

**Supplementary Table 2 Regulations on sulfur and its derivatives in OTC drugs&cosmetics**

| **Country/District** | **Intended use** | **Maximum concentrations** | |
| --- | --- | --- | --- |
| United States | OTC drugs | | 3-10% (Sulfur) (Acne products) |
|  |  |  | 0.6% (Micronized selenium sulfide)  (Antidandruff products) |
| China | Rinse-off products | | 1% (Selenium disulfide) 1.5% (Zinc pyrithione) |
|  | Leave-on products | | 11% (Sulfur) 0.1% (Zinc pyrithione) |
| European Countries | Rinse-off products | | 1% (Selenium disulfide)  (Antidandruff products) |
| Japan | - | | N/A |
| Korea | - | | N/A |

OTC= Over-the-Counter, N/A= Not applicable

**Supplementary Table 2 Regulations on sulfur and its derivatives in OTC drugs&cosmetics**

| **Products labeling** | **Data retrieved from** |
| --- | --- |
| For external use only Do not use on broken skin or large areas of the skin only to areas with acne | <https://www.fda.gov/drugs/historical-status-otc-rulemakings/rulemaking-history-otc-acne-drug-products> |
| N/A | <https://www.fda.gov/drugs/historical-status-otc-rulemakings/rulemaking-history-otc-dandruff-seborrheic-dermatitis-and-psoriasis-drug-products#Original> |
| Avoid contacts with eyes or damaged skin | <https://www.nmpa.gov.cn/hzhp/hzhpfgwj/hzhpgzwj/20151223120001986.html> |
| N/A | <https://www.nmpa.gov.cn/xxgk/ggtg/hzhpggtg/jmhzhptg/20210430162707173.html> |
| Avoid contacts with eyes or damaged skin | <https://eur-lex.europa.eu/legal-content/EN/ALL/?uri=CELEX%3A32009R1223> |
| N/A | <https://www.mhlw.go.jp/stf/seisakunitsuite/bunya/kenkou_iryou/iyakuhin/keshouhin/index.html> |
| N/A | <https://www.mfds.go.kr/eng/brd/m_60/view.do?seq=69887> |

OTC= Over-the-Counter, N/A= Not applicable
